# *Agrocybe cylindracea* Polysaccharides Ameliorate DSS-Induced Colitis by Restoring Intestinal Barrier Function and Reprogramming Immune Homeostasis via the Gut–Liver Axis

**DOI:** 10.3390/ijms26146805

**Published:** 2025-07-16

**Authors:** Aamna Atta, Muhammad Naveed, Mujeeb Ur Rahman, Yamina Alioui, Immad Ansari, Sharafat Ali, Eslam Ghaleb, Nabeel Ahmed Farooqui, Mohammad Abusidu, Yi Xin, Bin Feng

**Affiliations:** College of Basic Medical Science, Dalian Medical University, Dalian 116044, China; aaminaatta999@gmail.com (A.A.); naveed.uop10@gmail.com (M.N.); mujeeb166@gmail.com (M.U.R.); yalioui@outlook.fr (Y.A.); immadansari@outlook.com (I.A.); sharafat051@gmail.com (S.A.); eslamfahd059@gmail.com (E.G.); nabeel.farooqui99@yahoo.com (N.A.F.); mohammadabuseedo@gmail.com (M.A.)

**Keywords:** *Agrocybe cylindracea* polysaccharides, ulcerative colitis, dextran sulfate sodium, intestinal barrier, gut microbiota, TLR4/NF-κB pathway, immune homeostasis, gut–liver axis

## Abstract

Ulcerative colitis (UC) is a chronic inflammatory bowel disease driven by immune dysregulation, microbiota imbalance, and intestinal barrier dysfunction. Despite its global burden, effective therapies remain limited. This study explores the therapeutic potential of *Agrocybe cylindracea* polysaccharides (ACP) in a dextran sulfate sodium (DSS)-induced murine colitis model. High-performance liquid chromatography (HPLC)-characterized ACP was administered orally to BALB/c mice following colitis induction. ACP treatment significantly reduced Disease Activity Index (DAI) scores, preserved colon length, and restored intestinal barrier integrity by upregulating tight junction proteins. Mechanistically, ACP modulated immune homeostasis, suppressing pro-inflammatory cytokines (IL-17, IL-23, CRP) while enhancing anti-inflammatory mediators (IL-4, TGF-β). Furthermore, ACP inhibited hepatic TLR4/MyD88/NF-κB signaling, attenuated systemic inflammation, and reshaped gut microbiota composition by enriching beneficial taxa and reducing pathogenic *Bacteroides*. These findings demonstrate ACP multi-target efficacy in colitis, positioning it as a promising natural therapeutic for UC.

## 1. Introduction

Ulcerative colitis (UC) is a persistent inflammatory bowel disorder manifested by recurrent inflammation of the colonic mucosa, resulting in symptoms such as rectal bleeding, diarrhea, weight loss, and abdominal pain [1,2]. Often referred to as “green cancer” due to its chronic and debilitating nature, UC affects individuals regardless of age or gender and imposes a substantial economic burden on both individuals and society [3,4]. The global prevalence of UC is increasing, particularly in Western societies, with an annual incidence of 8.8–23.1 cases per 100,000 individuals in North America [5,6]. Despite advances in understanding its pathogenesis, the exact etiology of UC remains unclear, involving environmental factors, genetic predisposition, immune dysregulation, and imbalances in gut microbiota [7,8]. UC pathogenesis results from dysregulated immune homeostasis, characterized by the upregulation of pro-inflammatory cytokines, notably IL-17, IL-23, IL-6, TNF-α, and IL-1β, which leads to sustained colonic inflammation and epithelial barrier dysfunction [9]. These inflammatory mediators stimulate immunological cells, encompassing macrophages and T-helper lymphocytes, subsequently amplifying the inflammatory response [10]. In contrast, UC frequently downregulates anti-inflammatory mediators such as TGF-β and IL-10, which are crucial for preserving immunological homeostasis and facilitating mucosal tissue repair [11]. Intestinal epithelial barrier function preserved through tight junction proteins, including ZO-1, occludin, and claudin-1, becomes disrupted in UC, resulting in enhanced gut permeability and microbial translocation across the epithelium [12]. Recent investigations emphasize the pivotal contribution of intestinal microbiota to UC pathogenesis and advancement. Normal subjects maintain a heterogeneous and equilibrated microbial ecosystem, whereas UC individuals exhibit altered microbial composition, culminating in dysbiotic conditions [13]. Dysbiosis is characterized by a decrease in beneficial bacteria (e.g., *Lachnospiraceae* NK4A136 and *Lactobacillus*) and an increase in pathogenic species (e.g., *Parabacteroides* and *Bacteroides*), contributing to inflammation and barrier dysfunction [14]. Intestinal microbiota communicates with host immunological mechanisms via pattern recognition receptor (PRR) signaling cascades, particularly involving Toll-like receptors (TLRs), which detect microbial constituents such as lipopolysaccharides (LPSs) and initiate inflammatory cascades [15]. Dysbiosis in UC disrupts this interaction, leading to chronic inflammation and impaired mucosal healing [16]. Macrophages fulfill a pivotal role in UC pathogenesis, where an imbalance between inflammatory M1 macrophages identified by CD86 markers and regulatory M2 macrophages characterized by CD163 expression influences disease progression. M1 macrophages, activated by IFN-γ and LPS, release pro-inflammatory cytokines such as IL-6, TNF-α, and IL-12, facilitating inflammatory cascades and tissue damage. [17]. In contrast, M2 macrophages, induced by IL-4, produce anti-inflammatory cytokines, particularly IL-10 and TGF-β, promoting tissue repair and immune resolution [18]. In UC, this balance between M1 and M2 macrophages is disrupted, with excessive M1 activation and insufficient M2 response exacerbating colonic inflammation [19]. Targeting macrophage polarization to shift the balance from M1 to M2 is considered a potential treatment approach for UC [20]. Multiple investigations have demonstrated that ulcerative colitis affects essential organs, encompassing the spleen, kidneys, and liver. The spleen serves a crucial function in systemic immune responses and indicates overall immunological status [21,22]. Among these organs, the liver exhibits a significant correlation with inflammatory bowel disease (IBD) via the gut–liver axis [23]. The gut–liver axis is central to pathophysiology, as the liver receives direct portal venous drainage from the intestines, making it the primary target for translocated antigens and subsequent inflammatory activation. Intestinal barrier dysfunction, a hallmark of colitis, is characterized by reduced mucin and tight junction protein expression, which facilitates paracellular translocation of luminal bacteria, endotoxins, and LPS into systemic circulation through the activation of TLR4/MyD88/NF-κB signaling pathway, which is linked to the development of liver injury [24,25]. TLR4 activation by LPS triggers the recruitment of adaptor protein MyD88, which subsequently stimulates NF-κB, a primary regulator of inflammatory responses [26]. NF-κB upregulates the expression of pro-inflammatory cytokines, notably IL-6, IL-1β, and IL-18, which perpetuate inflammation and contribute to tissue injury in UC [27]. In the liver, activation of the TLR4/MyD88/NF-κB pathway by gut-derived LPS results in hepatic inflammation and fibrosis, underscoring the significance of the gut–liver axis in UC [28]. Quantifying the hepatic levels of TLR4, MyD88, and NF-κB in liver tissues through qPCR and Western blot analysis can provide insights into the molecular mechanisms driving systemic inflammation in UC [29]. Current pharmacological interventions for UC, including aminosalicylates, corticosteroids, and biologics, target inflammation reduction and remission maintenance; nevertheless, they frequently present substantial adverse effects and fail to guarantee long-term disease management [30]. First-line therapies, including 5-aminosalicylic acid (5-ASA), are effective for mild to moderate UC but can lead to adverse effects such as nephrotoxicity and hepatotoxicity with prolonged use [31]. Biologic agents targeting TNF-α and IL-12/23 are prescribed for moderate to severe UC, but they are expensive and may lose efficacy over time [32]. These limitations underscore the need for safer and more effective therapeutic strategies, especially those that address the underlying mechanisms of UC, such as immune dysregulation and gut microbiota imbalance [33]. Dietary interventions are critical in managing UC by modulating gut microbiota and intestinal immunity. Malabsorption, reduced intake, and excessive gastrointestinal losses in UC patients often lead to deficiencies in essential nutrients, including folic acid, iron, vitamin D, and vitamin B12, which impair mucosal healing and immune function [34]. Increasing the intake of nutrient-rich fruits and vegetables, along with targeted supplementation, can help restore nutritional balance and support mucosal integrity [35]. Additionally, dietary polysaccharides, particularly those derived from medicinal mushrooms, have shown promise in modulating gut microbiota and reducing inflammation in UC [36]. *A. cylindracea*, a commercially cultivated mushroom, is rich in bioactive polysaccharides that exhibit immunomodulatory, anti-inflammatory, and gut-protective effects [37]. *Pleurotus eryngii* has been demonstrated to regulate macrophage polarization, strengthen intestinal barrier integrity, and modulate gut microbiota composition [38]. *Agrocybe cylindracea* polysaccharides (ACP) also possesses antioxidant properties, reducing oxidative stress and protecting colonic tissue from damage [36]. These characteristics make ACP a promising candidate for UC therapy, particularly as a natural and cost-effective alternative to conventional treatments [39]. Based on existing research, *A. cylindracea* is widely cultivated and consumed for its nutritional and medicinal properties. The therapeutic potential and mechanisms of ACP in IBD, particularly its role in modulating the gut–liver axis, remain largely unexplored. This research aims to evaluate the impact of ACP on UC by modulating gut microbiota, regulating inflammatory and immune responses, and restoring intestinal barrier integrity. We hypothesize that ACP alleviates UC by downregulating inflammatory pathways, reducing systemic inflammation, and promoting homeostasis in the gut–liver axis.

## 2. Results

### 2.1. Chemical Characterization of ACP by HPLC

**Figure 1:**
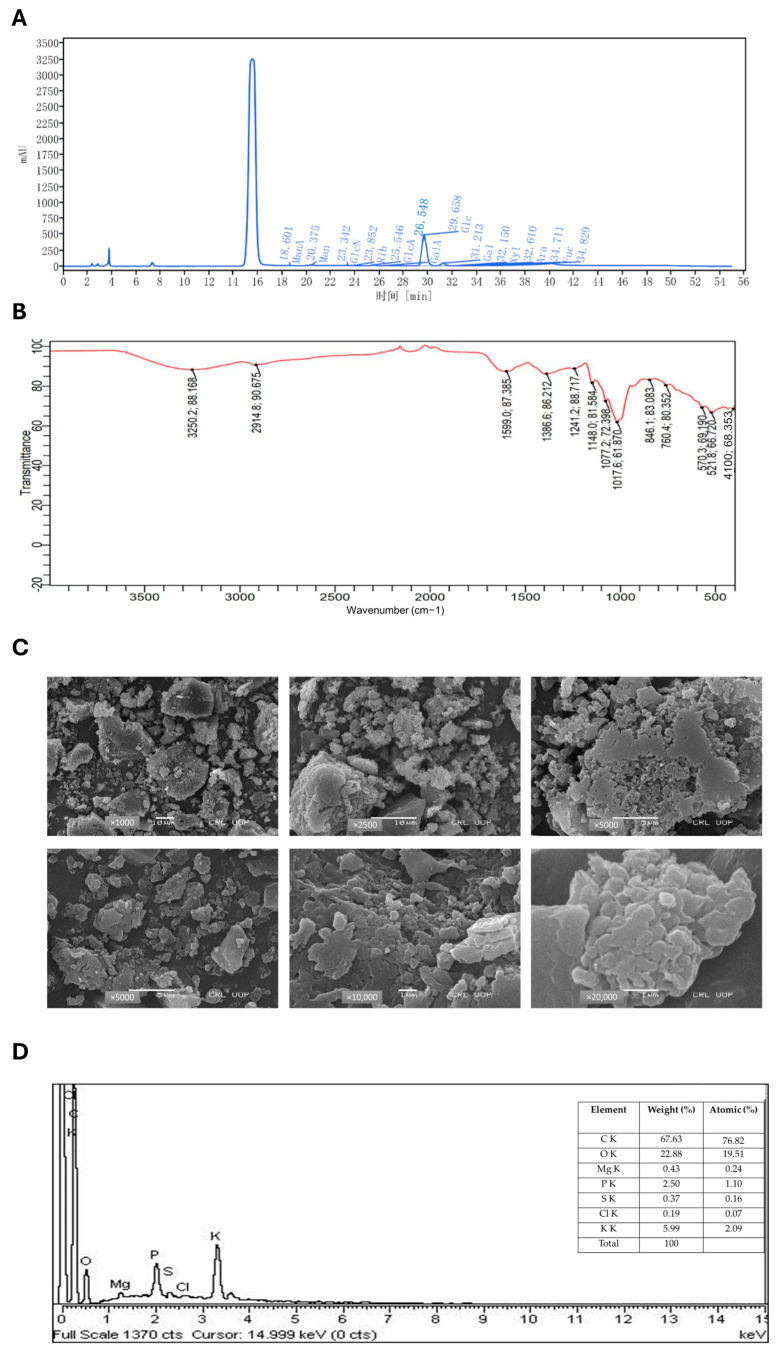
Characterization of *Agrocybe cylindracea* polysaccharides (ACP): (**A**) High-performance liquid chromatography (HPLC) analysis of crude ACP. (**B**) Fourier-transform infrared spectroscopy (FTIR) spectrum of the ACP sample displays distinct absorption peaks indicative of functional groups. (**C**) Scanning electron microscopy (SEM) images of the ACP at magnifications of 1000×, 2500×, 5000×, 10,000×, and 20,000× illustrate its surface morphology. (**D**) Energy dispersive X-ray (EDX) analysis of the ACP provides elemental composition data.

The crude ACP polysaccharide was obtained with a yield of 12% and contained 2.1% protein content. Quantitative assessment employing the phenol–sulfuric acid assay with D-glucose as a reference standard demonstrated a carbohydrate content of 45 mg/mL. The high-performance liquid chromatography (HPLC) analysis provided detailed monosaccharide composition (Figure 1A; Table 1), demonstrating that ACP is primarily composed of glucose (87.13%) and galactose (5.91%), with smaller amounts of glucuronic acid (2.72%) and mannose (1.56%). The polysaccharide also contained several minor components, including ribose (1.01%), galacturonic acid (0.85%), mannuronic acid (0.48%), fucose (0.27%), arabinose (0.04%), and xylose (0.02%). This diverse monosaccharide profile suggests the presence of complex polysaccharide structures in ACP, which may contribute to its biological activity. The high glucose content indicates that glucan-type polysaccharides are likely the major constituents of ACP.

### 2.2. FTIR Spectroscopy

Fourier-transform infrared spectroscopy (FTIR) analysis of ACP revealed characteristic absorption peaks between 400–4000 cm^−1^, identifying key functional groups (Figure 1B). The spectrum displayed a broad hydroxyl (O-H) stretch at 3250.2 cm^−1^, along with aliphatic C-H stretching at 2914.8 cm^−1^. A strong carbonyl (C=O) absorption appeared at 1599.0 cm^−1^, while peaks at 1386.6 cm^−1^ (C-H bending/aromatic vibrations), 1241.2 cm^−1^, and 1148.0 cm^−1^ (C-O stretching of esters/ethers) confirmed the polysaccharides complex structure. Additional fingerprint region signals (846.1 cm^−1^, 760.4 cm^−1^, 570.3 cm^−1^) suggested cyclic/aromatic components and the 410.0 cm^−1^ peak indicated potential metal–ligand interactions. Collectively, these results demonstrated ACP diverse functional groups, including hydroxyl, carbonyl, aliphatic, and aromatic moieties, characteristic of bioactive fungal polysaccharides.

### 2.3. SEM Microscopy

Scanning electron microscopy (SEM) revealed the morphological features of the crude polysaccharide sample (Figure 1C), which showed broken, uneven particles at low magnification. Higher magnification revealed smaller, fragmented pieces. These physical changes suggest the polysaccharide structure became more fragmented during processing.

### 2.4. EDX Spectroscopy

Energy dispersive X-ray (EDX) analysis was utilized to determine the elemental profile of the crude polysaccharide specimen (Figure 1D). This analytical technique detected characteristic X-ray emissions following electron beam exposure, revealing the sample’s fundamental organic constituents, including carbon (C) and oxygen (O), alongside various trace elements. The semi-quantitative analysis of X-ray peak intensities provided valuable information about elemental distribution and potential impurities. The EDX spectrum detected multiple biologically significant inorganic components, encompassing calcium (Ca), magnesium (Mg), silicon (Si), and potassium (K), alongside trace amounts of sulfur (S), phosphorus (P), and iron (Fe). These elements participate in critical fungal physiological processes such as cell wall maintenance, ionic homeostasis, and metabolic regulation. Additionally, the identification of mineral constituents, including calcium carbonate (CaCO_3_), silicon dioxide (SiO_2_), and magnesium oxide (MgO), indicated their integral function within the native polysaccharide framework. When integrated with SEM observations, these EDX results provided comprehensive evidence of the sample’s complex composition, demonstrating the intricate association between organic polysaccharides and inorganic constituents that collectively determine the structural and biological properties of the material. The presence of uronic acids and diverse monosaccharides, combined with these elemental findings, confirms the sample identity as a characteristic fungal-derived polysaccharide with potential bioactive properties.

### 2.5. ACP Attenuates Clinical Parameters and DAI Score

**Figure 2:**
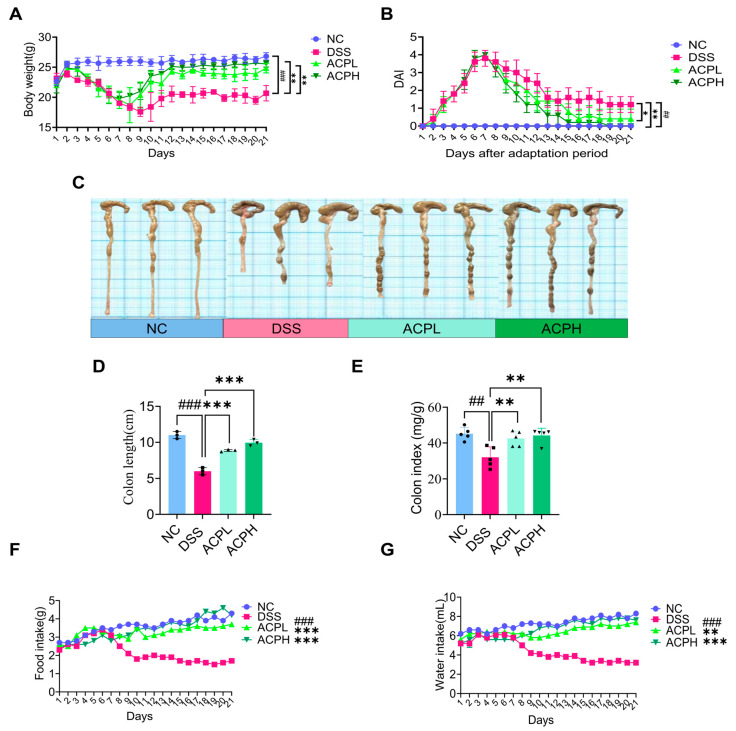
ACP alleviates colitis symptoms: (**A**) average weight change curves; (**B**) disease activity index scores; (**C**) colon length; (**D**) average colon length in cm; (**E**) colon index scores; (**F**) food intake; (**G**) water intake; results reflected as mean ± SD. ## *p* < 0.01 and ### *p* < 0.001 compared to NC; * *p* < 0.05, ** *p* < 0.01, and *** *p* < 0.001 compared to the dextran sulfate sodium (DSS) group.

The experimental colitis model was induced via oral delivery of 2.5% dextran sulfate sodium (DSS) in sterile water for 7 days, subsequently followed by a 14-day therapeutic intervention with ACP. Continuous assessment of clinical indicators, encompassing body weight alterations, rectal bleeding indices, and fecal consistency, was performed throughout both experimental phases. (Figure 2A,B). During DSS administration, all challenged groups developed characteristic ulcerative colitis symptoms, manifested by significant weight loss, severe diarrhea, and evident rectal bleeding, confirming successful disease induction. Following DSS withdrawal and initiation of ACP treatment, gradual improvement was observed in all treatment groups. The high-dose ACP group (ACPH) demonstrated particularly notable recovery, ultimately achieving a disease activity index (DAI) score of zero by the study endpoint, indicating complete resolution of clinical symptoms. In contrast, the normal control group maintained stable body weight and a DAI score of zero throughout the experimental period. Statistical analysis revealed significant differences in both body weight (DSS vs. NC: *p* < 0.001; ACPL/ACPH vs. DSS: *p* < 0.01) and DAI scores (DSS vs. NC: *p* < 0.01; ACPL vs. DSS: *p* < 0.05; ACPH vs. DSS: *p* < 0.01). These findings demonstrate that ACP, particularly at higher doses, effectively mitigates DSS-induced colitis symptoms, as evidenced by improved clinical parameters and reduced DAI scores.

### 2.6. ACP Ameliorates Colitis-Associated Morphological Changes

Evaluation of colitis severity demonstrated that ACP treatment significantly improved multiple disease parameters (Figure 2C–G and Figure S1A–E). Compared to the NC group, DSS-induced colitis caused pronounced pathological alterations, including reduced colon length (*p* < 0.001), decreased colon index (organ-to-body weight ratio, *p* < 0.01), diminished food intake (*p* < 0.001), lower water consumption (*p* < 0.001), and impaired weight gain (*p* < 0.001). ACP administration dose-dependently reversed these changes, with both low and high doses restoring colon length (both *p* < 0.001), colon index (both *p* < 0.01), food intake (both *p* < 0.001), and body weight (both *p* < 0.01) toward normal levels, while significantly improving water consumption (ACPL: *p* < 0.01; ACPH: *p* < 0.001). Systemic analysis revealed that DSS challenge increased spleen (*p* < 0.001) and liver (*p* < 0.001) indices while reducing small intestine (*p* < 0.05) and thymus (*p* < 0.01) weights, indicating widespread inflammatory effects. ACP treatment normalized these organ indices, with both doses significantly reducing spleen enlargement (ACPL: *p* < 0.05; ACPH: *p* < 0.01) and liver weight (ACPL: *p* < 0.01; ACPH: *p* < 0.001) while restoring small intestine (ACPL: *p* < 0.05; ACPH: *p* < 0.01) and thymus (ACPH: *p* < 0.05) weights. These comprehensive findings demonstrate that ACP treatment effectively mitigates colitis-associated pathology through local intestinal protection and systemic immunomodulatory effects, highlighting its therapeutic potential for managing inflammatory bowel disease.

### 2.7. ACP Attenuates Colitis-Associated Histopathological Damage

**Figure 3:**
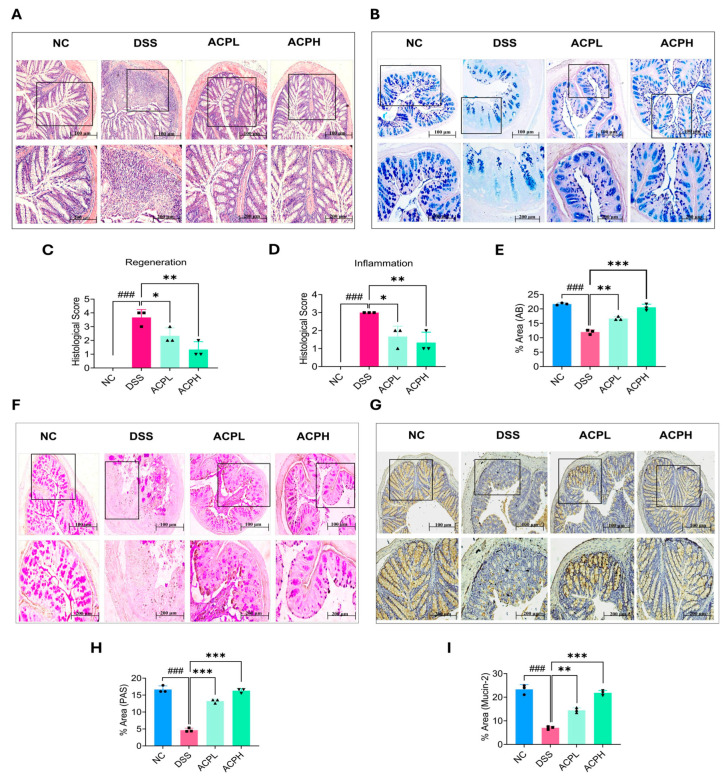
ACP mitigates DSS-induced histopathological damage in colon tissue: (**A**) H&E staining (upper: 10×, lower: 20×; scale bars: 100 μm, 200 μm) reveals histological alterations in colon tissue. (**B**) AB staining highlights acid mucins secreted by goblet cells (upper: 10×, lower: 20×; scale bars: 100 μm, 200 μm). (**C**) Histological scores for tissue regeneration and (**D**) inflammation quantify the extent of damage and recovery. (**E**) Quantification of AB staining demonstrates ACP-mediated enhancement of mucin expression. (**F**) PAS staining identifies neutral mucins in goblet cells. (**G**) IHC detects Mucin-2 protein expression in colon tissues (upper: 10×, lower: 20×; scale bars: 100 μm, 200 μm). (**H**) Quantifications of PAS staining and (**I**) IHC staining (Mucin-2) further validate the restorative effects of ACP. Data are presented as mean ± SD. ### *p* < 0.001 vs. NC; * *p* < 0.05, ** *p* < 0.01, and *** *p* < 0.001 vs. DSS group.

Histopathological evaluation of colon tissues using H&E staining revealed significant therapeutic effects of ACP on DSS-induced colitis (Figure 3A,C,D). The DSS group exhibited characteristic ulcerative colitis pathology, including extensive epithelial damage, goblet cell depletion, crypt abscess formation, and pronounced immune cell infiltration. In contrast, the NC group displayed intact epithelial architecture with abundant goblet cells and no inflammatory infiltrates. ACP treatment dose-dependently improved these pathological changes, with low and high doses reducing epithelial damage, restoring goblet cell populations, and diminishing inflammatory cell infiltration. The high-dose ACP group showed more pronounced restoration of colon architecture, approaching near-normal morphology. Quantitative histopathological scoring confirmed these observations, with the DSS group exhibiting severe inflammation compared to controls. Both ACP-treated groups demonstrated significantly reduced histological scores, with the high-dose group exhibiting significant improvement. These findings highlight the ability of ACP to preserve epithelial integrity, promote goblet cell recovery, and suppress inflammatory infiltration, underscoring its therapeutic potential for ulcerative colitis through tissue protection and repair mechanisms.

### 2.8. ACP Restores Mucosal Barrier Function in Colitis

The intestinal mucus layer, composed of neutral and acidic mucins secreted by goblet cells, forms a vital protective barrier for the colonic epithelium. In this study, we employed two complementary approaches to evaluate the effects of ACP on this barrier: Alcian Blue (AB) and Periodic Acid–Schiff (PAS) staining to assess overall mucus layer thickness and goblet cell numbers and Mucin-2 immunohistochemistry to specifically examine this crucial structural component of the mucus layer (Figure 3B,E–I). Histological analysis revealed that DSS-induced colitis caused severe damage to the mucus barrier, manifested by three key pathological changes: significant thinning of the mucus layer, reduction in goblet cell numbers, and decreased mucin production (Figure 3B,E,F,H). These findings were corroborated by Mucin-2 immunohistochemistry, which showed markedly reduced expression in DSS-treated animals compared to normal controls (Figure 3G,I). ACP treatment effectively reversed these alterations in a dose-dependent manner. Both low and high ACP doses improved mucus production, increased goblet cell populations, and restored mucus layer thickness, with the high-dose group (ACPH) demonstrating more pronounced therapeutic effects. Immunohistochemical analysis confirmed corresponding increases in Mucin-2 expression in ACP-treated groups, approaching levels observed in normal controls. These results validate that ACP protects against DSS-induced damage through multiple complementary mechanisms by stimulating goblet cell activity, enhancing mucin secretion (particularly Mucin-2), and promoting regeneration of the mucus layer architecture. The dose-dependent nature of these restorative effects highlights the therapeutic potential of ACP for colitis management, where reinforcement of the mucus barrier could help mitigate inflammation and prevent further epithelial damage.

### 2.9. ACP Modulation of CD68, CD86, and CD163 in Colitis

**Figure 4:**
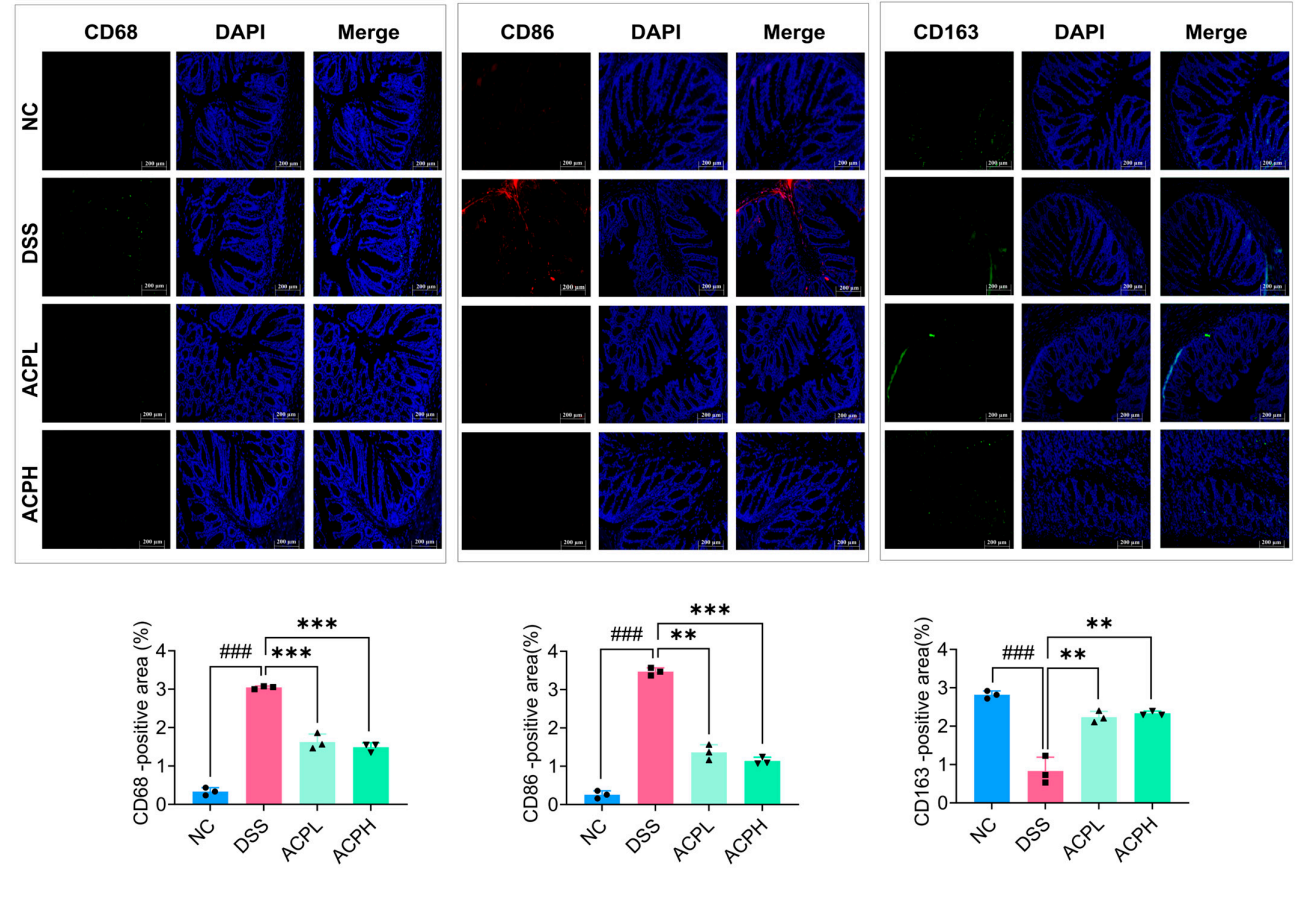
Immunofluorescent staining of macrophage markers in colon tissue. Immunofluorescent staining was performed to assess the expression of CD68-positive, CD86-positive, and CD163-positive markers in colon tissues across experimental groups. Images were captured at 20× magnification (scale bar: 200 μm), and quantification graphs for each marker were provided. Data are presented as mean ± SD. ### *p* < 0.001 vs. NC; ** *p* < 0.01 and *** *p* < 0.001 vs. DSS group.

Immunofluorescence examination of sigmoid colonic tissues demonstrated that DSS-induced colitis significantly upregulated pro-inflammatory macrophage markers (CD68, CD86) while downregulating the anti-inflammatory marker CD163 relative to NC (Figure 4). ACP treatment produced a dose-dependent shift in macrophages, with higher doses substantially decreasing CD68 and CD86 expression while increasing CD163 levels. These changes indicate the capacity of ACP to suppress inflammatory macrophage activation while promoting an anti-inflammatory phenotype, suggesting its therapeutic potential for rebalancing immune responses in colitis.

### 2.10. ACP Modulates Th-Cell Cytokine Balance in Colitis

**Figure 5:**
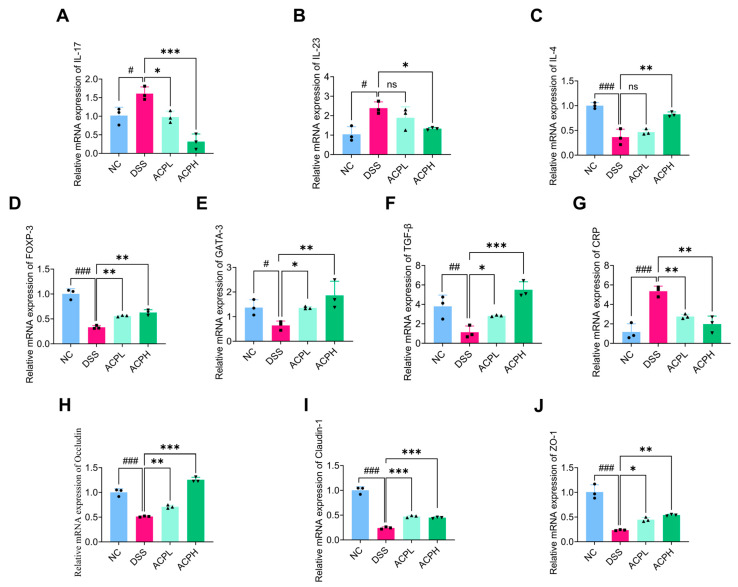
ACP regulates the balance of pro-inflammatory and anti-inflammatory mRNA expression levels of key markers in the colon: (**A**) IL-17; (**B**) IL-23; (**C**) IL-4; (**D**) FOXP3; (**E**) GATA3; (**F**) TGF-β; (**G**) CRP; (**H**) occludin; (**I**) claudin-1; (**J**) ZO-1. mRNA levels were normalized to GAPDH expression, and results are presented as mean ± SD. ns (not significant); * *p* < 0.05, ** *p* < 0.01, and *** *p* < 0.001 vs. DSS group; # *p* < 0.05, ## *p* < 0.01, and ### *p* < 0.001 vs. control group.

To assess T-helper cell responses, we analyzed cytokine mRNA expression in colonic tissues (Figure 5A–C). DSS-induced colitis significantly elevated pro-inflammatory mediators (IL-17, IL-23; both *p* < 0.05 vs. NC) while suppressing the anti-inflammatory cytokine IL-4 (*p* < 0.001). ACP treatment dose-dependently restored immune balance: both ACPL and ACPH reduced IL-17 (*p* < 0.05 and *p* < 0.001, respectively), with ACPH additionally suppressing IL-23 (*p* < 0.05) and significantly enhancing IL-4 expression (*p* < 0.01). These findings demonstrate the dual action of ACP in colitis, suppressing pathogenic Th17 responses while promoting protective Th2 activity.

### 2.11. Effect of ACP on Immune Modulation Markers in Colitis

Analysis of key immune markers revealed significant immunomodulatory effects of ACP in DSS-induced colitis (Figure 5D–G). Compared to NC, DSS-treated mice showed markedly reduced expression of anti-inflammatory markers FOXP3 (*p* < 0.001), GATA3 (*p* < 0.05), and TGF-β (*p* < 0.01), alongside elevated CRP (*p* < 0.001), indicating profound inflammatory dysregulation. ACP treatment dose-dependently reversed these effects, with both doses increasing FOXP3 (*p* < 0.01) and GATA3 (*p* < 0.05; *p* < 0.01), while ACPH showed stronger upregulation of TGF-β (*p* < 0.001 vs. *p* < 0.05 for ACPL). CRP levels were significantly reduced by both treatments (*p* < 0.01).

### 2.12. ACP Enhances Intestinal Barrier Function Through Tight Junction Regulation

Quantitative PCR analysis revealed that ACP significantly affects tight junction protein expression (Figure 5H–J). The DSS group demonstrated markedly reduced mRNA levels of occludin, claudin-1, and ZO-1 (all *p* < 0.001 vs. NC), which indicates severe impairment of intestinal barrier integrity. ACP treatment dose-dependently restored these critical proteins: occludin expression increased significantly (ACPL: *p* < 0.01; ACPH: *p* < 0.001 vs. DSS), as did ZO-1 (ACPL: *p* < 0.05; ACPH: *p* < 0.01) and Claudin-1 (both doses *p* < 0.001). These findings demonstrate the ability of ACP to repair epithelial barrier dysfunction in colitis by specifically upregulating key tight junction components, thereby reducing intestinal permeability.

### 2.13. Hepatic Cytokine Modulation by ACP Treatment

**Figure 6:**
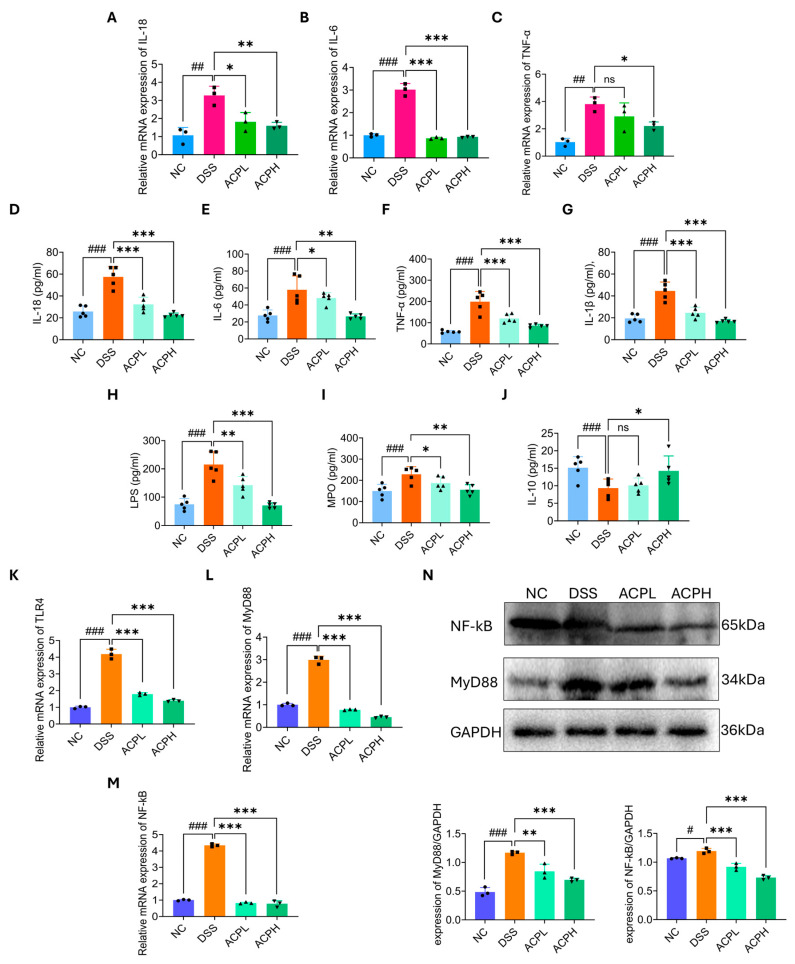
ACP attenuates pro-inflammatory signaling and cytokine production in hepatic tissues and serum: (**A**–**C**) mRNA expression of IL-18, IL-6, and TNF-α in hepatic tissue was significantly reduced by ACP treatment. (**D**–**J**) Cytokine levels (IL-18, IL-6, TNF-α, IL-1β, LPS, MPO, and IL-10) in liver tissue and serum indicate ACP-mediated suppression of inflammation. (**K**–**M**) ACP suppressed mRNA expression of TLR4, MyD88, and NF-κB in hepatic tissues. (**N**) Representative protein level and quantitative analysis of MyD88 and NF-κB normalized to GAPDH, demonstrating ACP-induced downregulation. Results are presented as mean ± SD. ns (not significant); * *p* < 0.05, ** *p* < 0.01, and *** *p* < 0.001 vs. DSS group; # *p* < 0.05, ## *p* < 0.01, and ### *p* < 0.001 vs. control group.

Analysis of hepatic mRNA expression revealed significant inflammatory changes in DSS-induced colitis (Figure 6A–C). IL-18 showed marked elevation in DSS mice (*p* < 0.01 vs. NC), which was significantly attenuated by both ACP doses (ACPL: *p* < 0.05; ACPH: *p* < 0.01). IL-6 exhibited the most pronounced increase (*p* < 0.001) and was strongly suppressed by both ACP treatments (both *p* < 0.001). TNF-α expression was elevated (*p* < 0.01) and showed dose-dependent reduction (ACPH: *p* < 0.05; ACPL: ns).

### 2.14. Hepatic Anti-Inflammatory Effects of ACP in DSS-Induced Injury

ELISA quantification of hepatic inflammatory mediators (Figure 6D–J) revealed that the DSS challenge induced profound inflammatory changes, with IL-18 showing the most dramatic elevation (*p* < 0.001 vs. NC). IL-6 levels were similarly increased (*p* < 0.001), followed by significant rises in TNF-α (*p* < 0.001) and IL-1β (*p* < 0.001). These cytokine disturbances were accompanied by substantial LPS accumulation (*p* < 0.001) and consequent MPO activity enhancement (*p* < 0.001), indicating endotoxin-mediated neutrophil infiltration. Concurrently, protective IL-10 production was severely compromised (*p* < 0.001). ACP treatment demonstrated dose-dependent therapeutic efficacy. Both ACPL and ACPH significantly decrease IL-18 (*p* < 0.001 vs. DSS). For IL-6, ACPL and ACPH showed significant suppression (ACPL: *p* < 0.05; ACPH: *p* < 0.01), while TNF-α reduction was stabilized (ACPL: *p* < 0.001; ACPH: *p* < 0.001). Both doses equally inhibited IL-1β (*p* < 0.001). The treatment also effectively reduced LPS burden (ACPL: *p* < 0.01; ACPH: *p* < 0.001) and MPO activity (ACPL: *p* < 0.05; ACPH: *p* < 0.01). Notably, ACPH partially restored IL-10 (*p* < 0.05), demonstrating comprehensive anti-inflammatory action.

### 2.15. ACP Modulates TLR4/MyD88/NF-κB Signaling

To investigate the systemic anti-inflammatory mechanisms of ACP, we analyzed the TLR4/MyD88/NF-κB pathway, a critical mediator of inflammatory responses in the gut–liver axis. Quantitative PCR revealed that both ACP doses significantly downregulated hepatic mRNA expression of TLR4, MyD88, and NF-κB compared to DSS-treated mice (*p* < 0.001 for all targets) (Figure 6K–M), demonstrating potent pathway inhibition. Western blot analysis further confirmed these findings at the protein level (Figure 6N). DSS challenge markedly increased MyD88 expression versus NC (*p* < 0.001), which was more effectively suppressed by ACPH (*p* < 0.001) than ACPL (*p* < 0.01). Similarly, DSS-induced phosphorylation of NF-κB p65 (*p* < 0.05) was significantly attenuated by both ACP treatments (*p* < 0.001), with ACPH showing greater efficacy. These coordinated reductions in both gene and protein expression of key inflammatory mediators substantiate ACP dose-dependent capacity to inhibit TLR4/NF-κB signaling, thereby mitigating systemic inflammation along the gut–liver axis in colitis.

### 2.16. Microbial Diversity Analysis of ACP Treatment in Colitis Model

**Figure 7:**
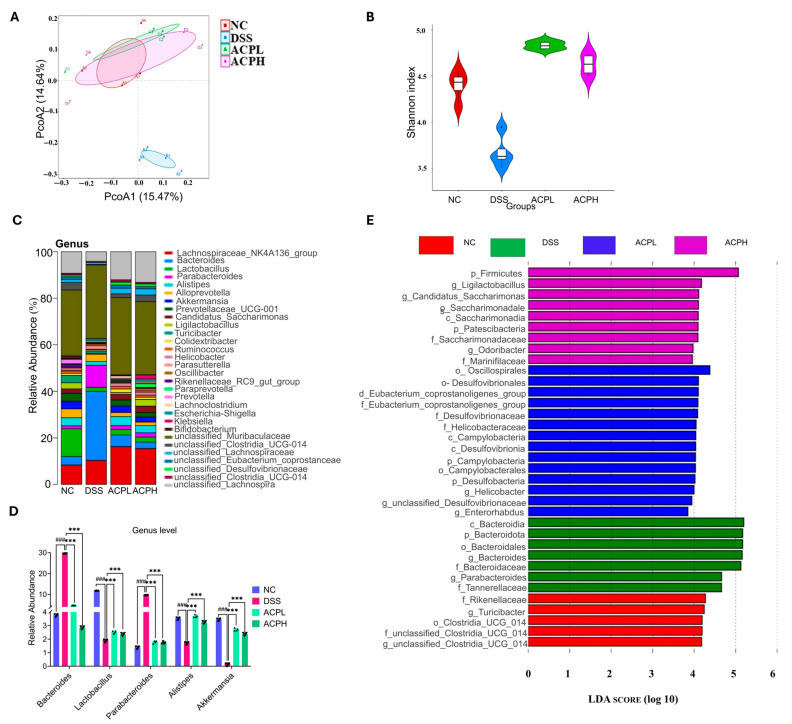
ACP mitigates DSS-induced gut dysbiosis: (**A**) Beta diversity analyses by Principal Coordinates Analysis (PCoA) to evaluate microbial community structure. (**B**) The alpha diversity, including the Shannon index, employ to assess within-sample taxonomic diversity. (**C**,**D**) Microbial diversity at the taxonomic level: Genus. (**E**) Linear Discriminant Analysis (LDA) scores (LDA > 4.0) highlight LEfSe key microbial biomarkers. Results reflected as mean ± SD. ### *p* < 0.001 compared to NC; *** *p* < 0.001 compared to the DSS group.

To assess the prebiotic impacts of ACP on intestinal microbial composition in DSS-induced colitis, we conducted 16S rRNA sequencing of the V3–V4 region from fecal samples. Beta diversity assessment using PCoA (Figure 7A), NMDS with weighted UniFrac distance, and PCA (Figure S2A,B) showed a clear separation between groups, with the DSS microbiota clustering distinctly from NC and ACP-treated groups progressively shifting toward the NC profile. Alpha diversity analysis confirmed these observations, demonstrating that the DSS group had significantly lower diversity Shannon index (Figure 7B) and species richness Ace and Chao1 indices (Figure S2C,D) than the NC group; at the same time, ACP supplementation dose-dependently restored these parameters, with the ACPH group showing more significant improvement than ACPL. Analysis of abundance rank curves (Figure S2E) and OTU rarefaction curves (Figure S2F) revealed that the DSS group exhibited the lowest microbial diversity, with curves stabilizing at fewer observed OTUs compared to both the NC group, which showed the greatest microbial diversity and OTU richness, and the ACP-treated groups, which displayed intermediate profiles. Taxonomic analysis across multiple hierarchical levels revealed significant alterations in intestinal microbiota composition following DSS-induced colitis and subsequent ACP intervention. At the phylum level (Figure S3A; Table S1), healthy controls (NC) maintained a balanced microbiome dominated by *Bacteroidota* and *Firmicutes* with minor *Verrucomicrobiota* representation (all *p* < 0.001 vs. DSS). ACP treatment dose-dependently restored microbial balance, with the high-dose group (ACPH) achieving near-physiological proportions of *Bacteroidota*, *Firmicutes*, and *Verrucomicrobiota* (all *p* < 0.001 vs. DSS). This restorative pattern persisted through finer taxonomic classifications. The class-level profile (Figure S3B; Table S2) of *Bacteroidia*, *Clostridia*, and *Bacilli* was severely disrupted by DSS (all *p* < 0.001 vs. NC). ACPH treatment effectively normalized these populations of *Bacteroidia*, *Clostridia*, and *Bacilli* (all *p* < 0.001 vs. DSS), demonstrating superior efficacy to the low-dose group (ACPL). Genus-level analysis (Figure 7C,D; Table S3) revealed DSS-induced depletion of beneficial taxa *Lactobacillus*, *Alistipes*, *and Akkermansia* and expansion of pathobionts *Bacteroides* and *Parabacteroides* (all *p* < 0.001 vs. NC). ACPH treatment specifically enriched protective genera *Lactobacillus*, *Alistipes*, *and Akkermansia* while suppressing disease-associated species, *Bacteroides* and *Parabacteroides* (all *p* < 0.001 vs. DSS), with ACPL exceeding effects. The coordinated restoration of microbial architecture across all taxonomic levels underscores the capability of ACP to counteract colitis-associated dysbiosis in a dose-dependent manner. LEfSe analysis (Figure 7E) identified key taxonomic differences: the NC group was dominated by the *Rikenellaceae* RC9 gut group, while the DSS group showed enrichment of *Bacteroides*, a marker of dysbiosis. The ACPL group demonstrated an increased abundance of beneficial taxa, including *Desulfovibrio* and *Eubacterium*, and the ACPH group was characterized by *Firmicutes* and *Ligilactobacillus* species. These findings collectively demonstrate that ACP treatment effectively counteracts DSS-induced dysbiosis by dose-dependently restoring both alpha and beta diversity while promoting beneficial bacterial taxa, supporting its potential as a therapeutic prebiotic for colitis management through gut microbiome modulation.

## 3. Discussion

In pathological conditions such as UC, wherein gut homeostatic disruptions precipitate hepatic complications, the gut–liver axis serves as a critical mediator of systemic inflammatory consequences. The importance of exploring novel therapeutic strategies targeting this axis is underscored by the intricate relationships between intestinal microbiota, immunological responses, and hepatic function. In the present investigation, we evaluated the potential therapeutic efficacy of ACP in a DSS-induced model, with particular emphasis on their modulatory effects on immune regulation, gut–liver axis equilibrium, and intestinal microbial ecology. Our findings demonstrate that ACP effectively alleviates colitis by modulating key pathways within the gut–liver axis, restoring intestinal barrier integrity, and suppressing systemic and hepatic inflammation. Notably, ACP administration was associated with the rebalancing of gut microbiota, leading to reduced dysbiosis and improved mucosal immunity. Furthermore, we observed a significant downregulation of pro-inflammatory cytokines in both intestinal and hepatic tissues, suggesting a broader immunomodulatory role of ACP beyond the gut.

ACP was extracted and subjected to comprehensive structural and compositional characterization using HPLC, FTIR, SEM, and EDX. HPLC analysis revealed that ACP consists of multiple monosaccharide components, including glucose, galactose, mannose, ribose, and glucuronic acid, which are commonly found in bioactive polysaccharides. ACP monosaccharide composition is mainly composed of glucose (Glc) and galactose (Gal), and this sugar composition is consistent with previous studies on ACP and other mushroom-derived polysaccharides, which strengthens the evidence supporting their potential biological activities [40]. FTIR analysis confirmed the presence of key functional groups, including aliphatic hydrocarbons, carbonyl compounds, and aromatic structures, which are characteristic of polysaccharides with known anti-inflammatory and immunomodulatory properties. These functional groups are critical for interactions with immune receptors and signaling pathways, potentially contributing to the therapeutic effects of ACP, and the results are aligned with the previous FTIR analysis of ACP [41]. Additionally, SEM analysis provided insights into the surface morphology of ACP, revealing a porous and irregular structure that may enhance its solubility and bioavailability. EDX further complemented this characterization by determining the elemental composition of ACP, confirming the presence of essential elements that contribute to its structural integrity and biological function [42]. The structural characterization of ACP aligns with recent findings on bioactive polysaccharides from edible mushrooms, which have been shown to reveal a wide range of beneficial properties, including anti-inflammatory, antioxidant, and immunomodulatory effects [43]. By providing detailed physicochemical analysis, our study extends the current understanding of ACP and demonstrates its potential as a treatment for colitis. These findings pave the way for further investigation into the mechanisms underlying the bioactivity of ACP and its possible applications in IBD management. The DSS-induced model was established to closely mimic morphological and clinical features of human UC, providing a well-recognized platform for evaluating potential therapeutic interventions. DSS administration resulted in significant weight loss, increased DAI, shortening of the colon, and notable alterations in food and water intake, all of which are hallmark indicators of colitis progression. These pathological alterations align with earlier research and reinforce the reliability of the DSS model in studying colitis-associated inflammation and gut barrier dysfunction [44,45]. Notably, ACP treatment significantly mitigated these adverse effects, leading to improvements in body weight retention, restoration of colon length, normalization of food and water intake, and other organ indices. These findings suggest that ACP effectively counteracts the deleterious impact of DSS-induced colitis, potentially by modulating inflammation, enhancing gut barrier integrity, and restoring homeostasis within the gut microenvironment. Similar therapeutic effects have been observed with polysaccharides derived from *Cantharellus cibarius* and *Dictyophora indusiata*, both of which have been shown to alleviate colitis symptoms by regulating immune responses and modifying gut microbiota composition [46,47,48]. The improvement in clinical parameters following ACP administration highlights its strong therapeutic potential in colitis management. Given the emerging evidence supporting the role of gut microbiota in colitis progression and recovery, our findings align with recent studies demonstrating the efficacy of polysaccharides in mitigating colitis severity through microbiota modulation and immune regulation [49,50]. These results underscore the importance of exploring natural bioactive compounds as promising candidates for colitis treatment, particularly in the context of gut microbiota-driven interventions. When colon tissue from DSS-induced colitis mice was examined histologically, it showed significant mucosal damage with crypt destruction, goblet cell depletion, epithelial erosion, and a noticeable infiltration of inflammatory cells into the lamina propria. These pathological changes, which are characteristic of ulcerative colitis, lead to persistent inflammation and compromised intestinal barrier function. As demonstrated by the restoration of crypt architecture, increased goblet cell density, and decreased inflammatory cell infiltration, ACP treatment markedly improved these histological abnormalities. Because goblet cells secrete mucins that preserve the integrity of the protective mucus layer, their preservation is especially important. We used immunohistochemical staining for Mucin-2 in addition to histochemical staining with AB and PAS to further evaluate the integrity of the mucus layer.

These analyses confirmed that ACP treatment effectively restored mucus production, reinforcing the intestinal barrier and preventing further epithelial damage. The restoration of the mucus layer is a key factor in maintaining gut homeostasis, as its depletion is strongly associated with increased susceptibility to inflammation and microbial translocation [51,52]. Our findings are in agreement with recent studies demonstrating that polysaccharides derived from *Scorias spongiosa* and *Gracilaria lemaneiformis* promote colonic tissue repair and attenuate inflammation in colitis models by enhancing mucosal integrity and modulating immune responses [49,53]. The histological improvements observed in our study underscore the tissue-repairing effects of ACP, which appear to be mediated through the restoration of the mucus layer, reinforcement of the intestinal barrier, and decline of inflammatory cell infiltration. These results further support the evidence, highlighting the therapeutic efficacy of mushroom-derived polysaccharides in colitis treatment, reinforcing their role as promising candidates for gut health interventions [54]. Immunofluorescence staining of colon tissue revealed that DSS-induced colitis resulted in a pronounced upregulation of pro-inflammatory macrophage markers, specifically CD68 and CD86, which are associated with M1 macrophage polarization and heightened inflammatory responses. Concurrently, there was a significant decrease in the expression of the anti-inflammatory macrophage marker CD163, indicating a shift towards a pro-inflammatory immune environment. However, ACP treatment effectively reversed these changes, enhancing a shift in macrophage polarization toward the anti-inflammatory M2 phenotype. This shift suggests that ACP not only mitigates inflammatory processes but also enhances tissue repair and immune regulation, key aspects of resolving colitis pathology [51]. These results were validated by additional mRNA analysis of colon tissue, which revealed that ACP substantially downregulates the expression of pro-inflammatory mediators, including TNF-α, IL-17, IL-18, and IL-23, which are typically overexpressed in colitis and facilitate tissue destruction and immunological dysregulation. Conversely, anti-inflammatory cytokines such as IL-4 and TGF-β, as well as immune regulatory markers like FOXP3 and GATA3, which are linked to the induction and maintenance of Tregs, were markedly upregulated upon ACP treatment. Tregs are critical in suppressing excessive inflammation and maintaining immune homeostasis, particularly in inflammatory diseases like colitis. These results are in line with new research showing that polysaccharides can alter immune responses by increasing Treg activity and reducing pro-inflammatory cytokine production [51,55,56]. The immunomodulatory effects of ACP align with the emerging literature on the role of polysaccharides in regulating immune responses, promoting immune tolerance, and restoring balance in immune homeostasis in colitis models [57]. Our study extends this body of work by demonstrating that the therapeutic effects of ACP are mediated, at least in part, through modulation of the gut–liver axis, suggesting that the effects of ACP are not confined to the gut but also influence systemic immune regulation and hepatic inflammation. This illustrates the broader therapeutic applications of ACP in colitis management and potentially other inflammatory conditions linked to gut–liver axis disruption. The synthesis of essential tight junction proteins, including occludin, claudin-1, and ZO-1, was markedly diminished in DSS-induced colitis, as determined by qPCR examination of colonic tissue. This reduction is indicative of increased intestinal permeability, a hallmark feature of colitis that contributes to compromised mucosal integrity, gut inflammation, and microbial translocation. These alterations in tight junction protein expression are consistent with the pathophysiology of colitis, where disruptions in the intestinal barrier allow for the infiltration of pathogenic microorganisms and immune cells, exacerbating the inflammatory response. Nonetheless, ACP treatment successfully increased the expression of these tight junction proteins, indicating that it protects the intestinal barrier. By reinforcing the tight junctions, ACP helps to restore intestinal permeability to near-normal levels, potentially preventing further damage and promoting mucosal healing. This restoration of tight junction integrity is critical for maintaining gut homeostasis and preventing the exacerbation of inflammation. Our observations align with recent investigations demonstrating that polysaccharides from *Hericium erinaceus* and *Gracilaria lemaneiformis* can strengthen intestinal barrier integrity by enhancing tight junction protein expression in experimental colitis models [49,54,58]. The protective mechanisms of ACP appear to be partially facilitated by its ability to regulate immunological and intestinal microbial processes, which are fundamental for maintaining intestinal barrier integrity. ACP contributes to preserving the intestinal barrier’s structural and functional integrity by affecting the gut microbiota makeup and encouraging a more balanced immune response. These findings add to the increasing amount of research on polysaccharides’ potential as a therapeutic agent for colitis and for enhancing intestinal barrier function [46]. ACP exhibits promise as a novel therapeutic approach for the treatment of colitis and other gastrointestinal disorders marked by barrier dysfunction by boosting epithelial barrier integrity and restoring tight junction protein expression. Analysis of liver tissue from DSS-induced colitis mice revealed elevated production of pro-inflammatory cytokines, such as IL-1β, IL-6, and TNF-α, along with upregulated LPS and MPO levels. These changes are indicative of systemic inflammation and hepatic immune activation, which are commonly observed in colitis-associated liver inflammation. The elevated hepatic LPS levels further indicate the translocation of microbial components from the inflamed intestine into the systemic circulation, contributing to hepatic immune activation and inflammatory responses. However, ACP treatment notably decreased the levels of these inflammatory markers, highlighting their anti-inflammatory properties. This decrease implies that ACP not only reduces intestinal inflammation but also, by modifying the gut–liver axis, protects distant organs like the liver. Western blot analysis and qPCR confirmed that ACP suppressed the activation of the TLR4/MyD88/NF-κB signaling cascade in hepatic tissue, which elucidated the underlying mechanism. The synthesis of pro-inflammatory cytokines and other inflammatory mediators is recognized to be driven by this pathway, which serves as a primary mediator of hepatic inflammation [59,60]. ACP seems to reduce hepatic inflammation and aid in the resolution of systemic inflammation in colitis by blocking this pathway. These results are aligned with recent research that demonstrates how polysaccharides from various natural sources can reduce hepatic inflammation by controlling the gut–liver axis, which is a critical connection between gut microbiota and immune responses in the liver [61]. The potential of ACP as a treatment for liver inflammation linked to colitis is highlighted by its capacity to suppress the TLR4/MyD88/NF-κB pathway in the liver. Additionally, these results align with recent studies that highlight the significant role of the gut–liver axis in mediating systemic inflammation, especially in inflammatory bowel diseases such as colitis [62]. By targeting this axis, ACP presents a promising approach for managing not only colonic inflammation but also its systemic effects, including liver involvement. The prebiotic impacts of ACP on intestinal microbial composition and diversity were assessed in a DSS-induced model utilizing 16S rRNA sequencing analysis. The DSS group exhibited the lowest microbial diversity, as evidenced by rarefaction curves and alpha diversity indices (Ace, Chao1, Simpson, and Shannon), reflecting severe gut dysbiosis. In contrast, ACP supplementation significantly restored microbial diversity in a dose-dependent manner, with the ACPH group displaying microbial profiles closely resembling those of the NC group. Beta diversity analysis further demonstrated that ACP treatment shifted microbial composition toward a healthier state, with NMDS and PCoA plots showing distinct clustering of the ACP-treated groups closer to the NC group. Taxonomic analysis revealed that DSS-induced colitis resulted in an increased abundance of *Bacteroides acidifaciens* and a depletion of beneficial microbial groups, including *Lachnospiraceae NK4A136* and *Lactobacillus*. However, ACP supplementation reversed these trends, enriching beneficial genera such as *Desulfovibrio*, *Lachnospiraceae NK4A136*, *Alistipes*, and *Ligilactobacillus*, indicating its potential to modulate gut microbiota toward a balanced state and short-chain fatty acid (SCFA) production. These findings align with prior studies demonstrating the protective benefits of mushroom-derived polysaccharides against DSS-induced colitis. For instance, *Dictyophora indusiate* polysaccharides (DIPs) alleviated colitis symptoms by restoring gut epithelial integrity, reducing oxidative stress, and modulating microbial diversity [63,64,65]. Similarly, *Ganoderma lucidum* polysaccharides (GLPs) improved survival rates, reduced body weight loss, and suppressed pro-inflammatory cytokines (IL-1β, IL-6, and TNF-α) in DSS-treated mice, highlighting their immunomodulatory effects. GLP improves gut microbiota by increasing beneficial bacteria *Akkermansia* and SCFA production [66]. Furthermore, an acidic polysaccharide from Phellinus linteus (PLAP) ameliorated colitis manifestations by suppressing pro-inflammatory mediators, promoting anti-inflammatory cytokines, and reestablishing intestinal microbiota diversity [67]. Furthermore, polysaccharides from *Mytilus coruscus* and *Cordyceps sinensis* modulated the gut microbiota by lowering the levels of *Bilophila* while increasing beneficial genera such as *Dehalobacterium*, *Alistipes*, and *Coprococcus*, accompanied by enhanced SCFA production [68,69]. A similar effect was observed with *Pleurotus tuber-regium mycelium* polysaccharides, which regulated inflammatory cytokines, oxidative stress, and microbial balance [70]. Collectively, these results highlight the therapeutic potential of mushroom-derived polysaccharides, including ACP, in counteracting DSS-induced dysbiosis and restoring gut microbial homeostasis. This study demonstrates that ACP mitigates DSS-induced colitis through modulation of the gut–liver axis, restoring intestinal barrier function, and suppressing systemic and hepatic inflammation. The therapeutic efficacy of ACP is accompanied by suppression of the TLR4/MyD88/NF-κB cascade, modulation of intestinal microbiota, and immunological regulation. These studies emphasize the potential of ACP as an innovative therapy for UC and other gut–liver axis-associated disorders. Although this investigation provides significant insights into ACP therapeutic effects on DSS-induced colitis in murine models, future research should address fundamental limitations. Supplementation of an only-ACP-treated group would provide baseline effect evaluation, and toxicological assessments would strengthen safety characterization. Future investigations incorporating metabolomics, functional validations, and systematic safety assessments will provide a more complete therapeutic profile of ACP in IBD management.

## 4. Materials and Methods

### 4.1. Chemicals and Reagents

The Black Poplar Mushroom *A. cylindracea* was sourced from Yunnan Congrong Economic and Trade Co., Ltd., Kunming, China. Among the reagents, RIPA lysis buffer was obtained from Beyotime Biotechnology (Shanghai, China), dextran sulfate sodium (DSS, MW: 36,000–50,000) from Yeasen Biotechnology (Shanghai, China), and a BCA protein quantification kit from Jiancheng Bioengineering Institute (Nanjing, China). ELISA detection kits were supplied by Jiangsu Meibiao Biotechnology Co., Ltd. (Yancheng, China), while Proteintech (Wuhan, China) furnished the primary antibodies. ZSGB BIO (Beijing, China) provided the HRP-conjugated secondary antibody and the DAB substrate chromogen system. A commercial fecal DNA extraction kit from MoBio Laboratories (Carlsbad, CA, USA) was utilized for nucleic acid isolation. The remaining chemicals and reagents were all analytical grade and purchased from reliable commercial suppliers.

### 4.2. Preparation of A. cylindracea Crude Polysaccharides (ACP)

Upon receipt, the fruiting bodies of *A. cylindracea* mushrooms were cleaned, washed with distilled water to remove debris, and dried in a hot air oven at 60 °C. Then, the dehydrated mushrooms were finely ground and sieved through a 0.42 mm mesh to ensure uniformity. Two successive extractions were performed on the powder, both at 80 °C: the first ddH_2_O-to-powder ratio of 1:30 (g/mL) for 4 h and the second at a ratio of 1:20 (g/mL) for 2 h. Proteins were eliminated from the extracted solution by treating it with 1.5% (*v*/*v*) TCA, and NaOH was used to bring the pH down to 7. A rotary evaporator condensed the supernatant after the mixture was centrifuged for ten minutes at 5000× *g*. Polysaccharides were precipitated by adding four times the volume of 95% ethanol and kept overnight at 4 °C. The resulting precipitated material was collected as ACP after the blend was centrifuged for 10 min at 5000× *g*, and the resulting supernatant was removed. The ACP was freeze-dried [71], and its protein content was measured. The yield of ACP (%) was calculated using the following formula: yield% = *W*_0_/*W*_1_ × 100, *W*_0_ is the weight of the extracted crude polysaccharide, and *W*_1_ is the weight of the mushroom powder used.

### 4.3. Carbohydrate Content and Monosaccharide Composition

The phenol–H_2_SO_4_ method was used to quantify the carbohydrate content, employing D-glucose for calibration [72]. The monosaccharide profile of the ACP was examined through HPLC.

### 4.4. FTIR

The freeze-dried ACP sample was analyzed and characterized using FTIR spectroscopy with a Shimadzu FTIR-4200 spectrometer (Tokyo, Japan), employing the approach outlined [73]. The spectrometer was configured to operate across a frequency range of 500–4000 cm^−1^. The analysis was conducted at a scanning velocity of 10 scans per second, with a resolution of 4 cm^−1^.

### 4.5. SEM with EDX

ACP morphology and elemental composition were analyzed using SEM and EDX (Model S-2600N, Hitachi, Tokyo, Japan). Freeze-dried powder samples were mounted on carbon tape, sputter-coated with gold, and imaged at 15 kV to examine structural features. Simultaneous EDX analysis detected emitted X-rays under electron beam exposure, identifying carbon and oxygen as primary elements along with trace components, confirming the polysaccharide’s organic nature. Semi-quantitative elemental concentrations were derived from X-ray peak intensities, with measurements performed at 15 kV.

### 4.6. Experimental Design and Animal Housing

Thirty-two male-specific, pathogen-free (SPF) BALB/c mice, aged 5–6 weeks and weighing 20 ± 2 g, were procured from Liaoning Changsheng Biotechnology Co., Ltd. The mice were randomly allocated into four groups (*n* = 8 per group): normal control (NC), DSS-induced colitis (DSS), *A. cylindracea* polysaccharide low dose (ACPL), and *A. cylindracea* polysaccharide high dose (ACPH). After a one-week adaptation period in a sterile environment (temperature: 22 ± 3 °C; humidity: 50 ± 5%; 12-h light/dark cycle), the mice received SPF-grade rodent chow (Shenyang Mao Hua Biotechnology Co., Ltd., Shenyang, China) and distilled water ad libitum. All experimental protocols adhered to institutional and national ethical standards and received approval from the Dalian Medical University Ethics Committee (Registration No. AEE24192).

### 4.7. Animal Modeling and Treatment Protocol

**Figure 8:**
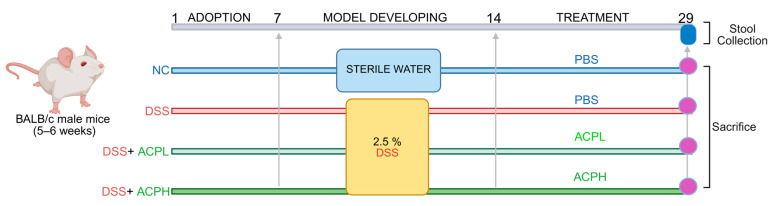
The experimental design was structured into three phases: adaptation, colitis induction, and treatment. Mice were divided into four groups (*n* = 8 per group): the NC group received regular sterile water and PBS orally for 14 days; the colitis model (DSS) group was administered 2.5% DSS in drinking water for 7 days to induce colitis, followed by PBS orally for 14 days; the low-dose treatment (ACPL) group received 2.5% DSS for 7 days, followed by low-dose ACP orally for 14 days; and the high-dose treatment (ACPH) group was given 2.5% DSS for 7 days, followed by high-dose ACP orally for 14 days. Fecal samples were collected on day 28, and all mice were sacrificed on day 29 for subsequent analyses.

To establish acute colitis, mice were administered 2.5% DSS in sterile drinking water for 7 days, subsequently followed by 14-day oral gavage interventions. The NC and DSS-induced model groups received PBS, while the treatment groups ACPL and ACPH received ACP (150 and 300 mg/kg, respectively)**.** A dose optimization investigation was performed to determine the therapeutic range of ACP. Mice received varying concentrations of ACP (50, 100, 150, 200, 300, and 400 mg/kg). The findings demonstrated that lower concentrations, 50 and 100 mg/kg, exhibited no substantial efficacy, while 150 and 300 mg/kg concentrations produced significant biological responses, justifying their selection for further experimentation. The highest concentration tested (400 mg/kg) demonstrated no additional advantages compared to the 300 mg/kg concentration. Consequently, 150 and 300 mg/kg were selected for subsequent investigations. Fecal specimens were preserved at −80 °C for microbiome examination. After 28 days, mice were euthanized to collect colon distal tissues (fixed in 4% formalin) for histopathology, along with blood, liver, and colon for molecular studies. The experimental design is illustrated in Figure 8.

### 4.8. Disease Activity Index

Throughout the study, colitis extent of severity was analyzed daily using the DAI, calculated based on the Murthy scoring system [74]. Body weight loss (1: 1–5%, 2: 5–10%, 3: 10–15%), stool consistency (0: normal, 1–2: loose stools, 3–4: diarrhea), and stool bleeding score (1: no detectable blood, 2: positive occult blood, 3: visible bleeding in stool) were all given 4-point ratings on the DAI. The final DAI score was computed using the formula DAI = 1/3 (Weight loss score + Stool consistency score + Bleeding score), providing a comprehensive evaluation of colitis progression. Body weight was recorded daily, while food and water consumption were documented every three days. The weights of the colon, spleen, small intestine, thymus, and liver were noted at the time of sacrifice, as was the length of the colon measured from the cecal base to the distal rectum. The following formula was used to determine the organ index (mg/g): Organ index (mg/g) = weight of the organ (mg)/weight of the mouse (g).

### 4.9. Colonic Histopathological Evaluation

Following sacrifice, distal colon specimens were harvested and preserved in 4% formalin fixation and maintained at room temperature for 24 h. Tissue samples were then sectioned, treated with H&E, and dehydrated through an ascending ethanol gradient and xylene solutions. The stained sections were cover-slipped with the mounting solution and sealed for subsequent microscopic examination. Slide preparations were examined using a microscope (Leica Microsystems, Wetzlar, Germany) in a blinded manner, and histological features, including tissue morphology, regeneration, and inflammation, were evaluated using a standardized scoring protocol (Table 2).

### 4.10. Evaluation of Mucus Epithelium Thickness and Goblet Cells

PAS and AB staining were used to assess the thickness of goblet cells and mucus epithelium in sigmoid colon tissue. Tissue slides were deparaffinized in xylene, rehydrated using a series of graded ethanols, and then exposed to periodic acid reagents for five minutes at room temperature to perform PAS staining. After rinsing with ultra-filtered water, slides were stained with Schiff reagent for 10 min, washed under running water, counterstained with hematoxylin, and fixed using neutral balsam (Solarbio, cat-G8590, Beijing, China). For AB staining (pH 2.5), slides were deparaffinized, hydrated, and immersed in 1% AB acetate solution for 10 min. Following oxidation in 1% PAS solution and rinsing, slides were stained with Schiff’s reagent, dehydrated, cleared, and mounted for analysis of randomly selected areas under a microscope and quantitative evaluation using ImageJ software (1.5.3).

### 4.11. IHC for Mucin-2

Mucin-2 expression in sigmoid colon tissue was assessed using IHC. Slides underwent deparaffinization, rehydration, and microwave antigen retrieval in citrate buffer (10 mM, pH 6.0). H_2_O_2_ inhibited endogenous peroxidase activity, and 5% BSA in PBS reduced nonspecific binding. Slides were kept overnight at 4 °C with a primary antibody against Mucin-2 (Proteintech, 27675-1-AP, 1:1000 dilution), followed by a secondary antibody and 3, 3′-diaminobenzidine (DAB) for visualization. After counterstaining with hematoxylin, slides were dehydrated, cleared, and mounted. Mucin-2-positive cells were analyzed in three randomly selected fields using a microscope, and a semi-quantitative assessment was performed using ImageJ software.

### 4.12. Immunofluorescent Staining for Macrophage Markers in Colon Tissue

Slides were first incubated at 65 °C for two hours to encourage tissue adhesion before immunofluorescence analysis of macrophage markers CD68, CD86, and CD163 in sigmoid colon tissue sections. Following xylene deparaffinization, the tissues were rehydrated using a series of graded ethanols (100%, 95%, 80%, 75%, and 50%) and ultra-pure water. Sections were heated in citrate buffer (10 mM, pH 6.0) using microwave irradiation to achieve antigen retrieval. After triple PBS washing, the sections were blocked with 5% bovine serum albumin (BSA) in PBS and incubated at 4 °C overnight with primary antibodies against CD68 (1:500, Proteintech, 28058-1-AP), CD86 (1:2000, Proteintech, 13395-1-AP), and CD163 (1:500, Proteintech, 16646-1-AP). Following primary antibody exposure, the sections were washed with PBS and incubated for one hour at room temperature with FITC-conjugated goat anti-rabbit secondary antibody (Proteintech). The nuclei were counterstained with DAPI for five minutes after additional PBS washes. To investigate the localization and expression patterns of macrophage markers in colonic tissue, the slides were cover-slipped in a DAPI-containing medium and examined under a fluorescence microscope in a blinded manner, and quantitative assessment was performed using ImageJ software.

### 4.13. Therapeutic Impact of ACP on DSS-Induced Colitis and Gut–Liver Axis at the mRNA Level

TLR4/MyD88/NF-κB inflammatory pathway, a crucial signaling cascade that connects gut inflammation to hepatic responses, was the focus of an analysis of colon and liver tissue samples to evaluate the therapeutic effect of ACP on DSS-induced intestinal inflammation and investigate the molecular mechanisms of colitis and its systemic effects on the gut–liver axis. The intestinal barrier integrity and immune regulation were evaluated in the colon by measuring the expression of inflammatory markers IL-17, IL-23, and IL-4, immune regulatory markers FOXP3, GATA3, TGF-β, and CRP, and tight junction proteins occludin, claudin-1, and ZO-1. The expression of inflammatory markers TNF-α, IL-18, and IL-6 and inflammatory pathways TLR4, MyD88, and NF-κB were investigated in the liver to investigate systemic inflammatory responses and hepatic involvement in colitis. Utilizing TRIzol reagent (Thermo Fisher Scientific, Waltham, MA, USA), total RNA was extracted from distal colon and liver tissues, measured using a NanoDrop ND-1000 spectrophotometer, and then stored at −80 °C. cDNA synthesis was performed using 1 µg of RNA with the HiScript II Q RT SuperMix for qPCR (Vazyme Biotech Co., Ltd. Nanjing, China), following the manufacturer’s protocol. A Bioer LightGene 9600 analyzer (Hitech, Hangzhou, China) equipped with a ChamQ SYBR qPCR MasterMix was used to perform gene expression analysis. The PCR protocol included an initial denaturation step at 95 °C for 10 min, followed by 40 cycles of 95 °C for 25 s and 60 °C for 1 min. The 2^−ΔΔCt^ method was employed to determine relative gene expression, which was subsequently normalized to the housekeeping gene β-actin and statistically evaluated using GraphPad Prism 10.2.3 and the Gene 9660 system, Rest2009 software. Table S4 contains primer sequences.

### 4.14. Evaluation of Hepatic Inflammation: LPS, MPO Activity, and Cytokine Levels

To evaluate the equilibrium between pro- and anti-inflammatory mediators, the concentrations of essential cytokines, including IL-18, IL-1β, TNF-α, IL-6, LPS, MPO, and IL-10, were quantified in serum and hepatic tissue homogenates. For tissue analysis, 100 mg of hepatic tissue was homogenized to 900 μL of PBS on ice and centrifuged at 3500× *g* for 15 min at 4 °C, and the resulting supernatant was collected and preserved at −80 °C until subsequent analysis. For biomarker assessment from serum, blood specimens were centrifuged for 20 min at 3000× *g*, followed by clotting for 20 min at room temperature [75]. The serum supernatant was subsequently aliquoted and stored at −80 °C. ELISA kits (Jiangsu Meibiao Biotechnology Co., Ltd.) were utilized to measure LPS concentrations, MPO activity, and cytokine levels. A colorimetric reaction commenced by adding 50 μL of chromogen A and 50 μL of chromogen B, followed by plate incubation at 37 °C for 15 min under dark conditions. The reaction was halted by adding 50 μL of stop solution, and absorbance was measured at 450 nm. Prior to analysis, ELISA reagents were equilibrated at room temperature for 30 min. A 50 μL volume of standard reference was dispensed into designated wells, while test specimens were loaded into corresponding wells. The plate was subsequently incubated at 37 °C for 60 min. Concentrations were calculated using a standard curve.

### 4.15. Western Blot Analysis

Proteins were extracted using RIPA lysis buffer and subsequently separated on gradient SDS-PAGE gels with an 8–12% gradient. Following electrophoresis, they were transferred onto a PVDF membrane using a semi-dry transfer system. The blotting membrane was incubated with a blocking buffer containing 5% skimmed milk in TBS for 2 h at room temperature, followed by three washes with TBST (TBS containing 0.1% Tween 20). The membrane was probed with primary antibodies MyD88 (1:1000, cat no. 29946-1-AP (Proteintech, Rosemont, IL, USA), NF-κB (1:1000, cat no. bs-0465R (Bioss, Woburn, MA, USA), and GAPDH (1:10,000, cat no. 10494-1-AP (Proteintech, Rosemont, IL, USA) as a loading control diluted in TBST overnight at 4 °C. After thorough washing, the detection was performed using HRP-conjugated secondary antibody (1:5000, catalog no. SA00001-2 (Proteintech, Rosemont, IL, USA) for 1 h at room temperature. Following additional washes, visualization was achieved using an ECL chemiluminescence kit on an automated imaging system (Imager-Bio-Rad, Bio-Rad Laboratories, Inc., Hercules, CA, USA). Densitometric analysis was conducted using ImageJ software.

### 4.16. Fecal Microbial DNA Isolation and 16S rRNA Gene Sequencing

Fecal microbial DNA was extracted from murine stool specimens utilizing the PowerMax DNA Isolation Kit (MoBio Laboratories, Carlsbad, CA, USA). Specimens (200–500 mg) were treated with lysozyme and a chaotropic salt solution to disrupt cellular walls and membranes, followed by protein degradation with protease. DNA was purified via spin-column technology, quantified using a NanoDrop ND-1000 spectrophotometer (Waltham, MA, USA), and assessed by 1% agarose gel electrophoresis. The 16S rRNA V3–V4 region was amplified using primers 341f (CCTACGGGAGGCAGCAG) and 518r (ATTACGCGGCTGCTG) under standard PCR conditions and sequenced on the Illumina NovaSeq 6000 platform (Sangon Biotech, Shanghai, China). Bioinformatics and microbial analysis were performed, and data were analyzed using QIIME. Alpha diversity indices (Chao1, Shannon, Ace, Simpson) assessed species richness and evenness, while Beta diversity was evaluated using weighted UniFrac distances. Microbial community structures were visualized via PCoA, PCA, and NMDS. Differential taxa were identified using LDA to determine key biomarkers, providing insights into gut microbiota composition and dynamics.

### 4.17. Biostatistical Analysis

GraphPad Prism 10.2.3 was used to carry out the biostatistical analysis. Standard one-way ANOVA with Tukey’s post hoc test for multiple comparisons was employed to establish statistical significance. Data were expressed as mean ± SD (SD, *n* ≥ 3), with statistical significance defined at *p* < 0.05. The Kruskal–Wallis test was utilized for group comparisons in 16S rRNA sequencing analysis. The Mann–Whitney U test was applied to evaluate OTU and phenotypic data. These approaches ensured robust and accurate statistical evaluation of the experimental results.

## 5. Conclusions

This study identifies ACP as a novel therapeutic agent for ulcerative colitis, with dual benefits for gut and liver health. ACP promotes colonic healing by restoring epithelial barrier proteins (ZO-1, occludin) and rebalancing mucosal immunity, suppressing pro-inflammatory Th17 responses (IL-17/IL-23) while enhancing anti-inflammatory Treg activity (TGF-β/IL-10). Notably, ACP modulates the gut–liver axis by enriching beneficial gut bacteria (*Lachnospiraceae*, *Clostridia*) and reducing liver inflammation via TLR4/NF-κB pathway inhibition. To our knowledge, this is the first report of a mushroom polysaccharide simultaneously targeting gut barrier repair, immune homeostasis, and systemic inflammation in colitis. While preclinical results are promising, clinical trials are needed to validate ACP efficacy in humans. These findings support the potential of fungal polysaccharides as dietary interventions for inflammatory bowel disease.

## Figures and Tables

**Table 1:** The monosaccharide composition of *Agrocybe cylindracea* crude polysaccharides.

Components	Concentration (mg/kg)	Percentage (%)
Mannuronic acid	1521.43	0.48
Mannose	4964.29	1.56
Ribose	3221.43	1.01
Glucuronic acid	8650	2.72
Galacturonic acid	2707.14	0.85
Glucose	276,950	87.13
Galactose	18,782.14	5.91
Xylose	75	0.02
Arabinose	128.57	0.04
Fucose	850	0.27

**Table 2:** Histopathological score: colon tissues were examined for inflammation and regeneration.

	Score	Significance
Regeneration	4	No tissue repair
	3	Surface epithelium not intact
	2	Regeneration with crypt depletion
	1	Almost complete regeneration
	0	Complete regeneration or normal tissue
Inflammation	3	Severe
	2	Moderate
	1	Slight
	0	None

## Data Availability

The raw data supporting the conclusions of this article will be made available by the authors upon request.

## Supplementary Materials

The following supporting information can be downloaded at: https://www.mdpi.com/article/10.3390/ijms26146805/s1.
