# Supplementary material for: Agrocybe cylindracea Polysaccharides Ameliorate DSS-Induced Colitis by Restoring Intestinal Barrier Function and Reprogramming Immune Homeostasis via the Gut–Liver Axis
Source: Int J Mol Sci. 2025 Jul 16;26(14):6805. doi: 10.3390/ijms26146805 (PMC12295746; doi:10.3390/ijms26146805)
Supplement: Supplementary file 1 [file ijms-26-06805-s001.zip › ijms-3686097-supplementary.pdf]

Agrocybe cylindracea Polysaccharides Ameliorate DSS-Induced Colitis by Restoring Intestinal Barrier Function and Reprogramming Immune Homeostasis via the Gut-Liver Axis

Aamna Atta , Muhammad Naveed , Mujeeb Ur Rahman , Yamina Alioui , Immad Ansari, Sharafat Ali, Eslam Ghaleb , Nabeel Ahmed Farooqui , Mohammad Abusidu , Yi Xin [1]*, Bin Feng [1]*

College of Basic Medical Science, Dalian Medical University, Dalian 116044, China.
aaminaatta999@gmail.com (A.A.); naveed.uop10@gmail.com (M.N.); Mujeeb166@gmail.com (M.U.R.); yalioui@outlook.fr (Y.A.); Immadansari@outlook.com (I.A.); sharafat051@gmail.com (S.A.); eslamfahd059@gmail.com (E.G.); nabeel.farooqui99@yahoo.com (N.A.F.); mohammadabuseedo@gmail.com (M.A.).
* Correspondance: Email : binfeng@dmu.edu.cn (B.F); xinyi412@dmu.edu.cn (Y.X.)

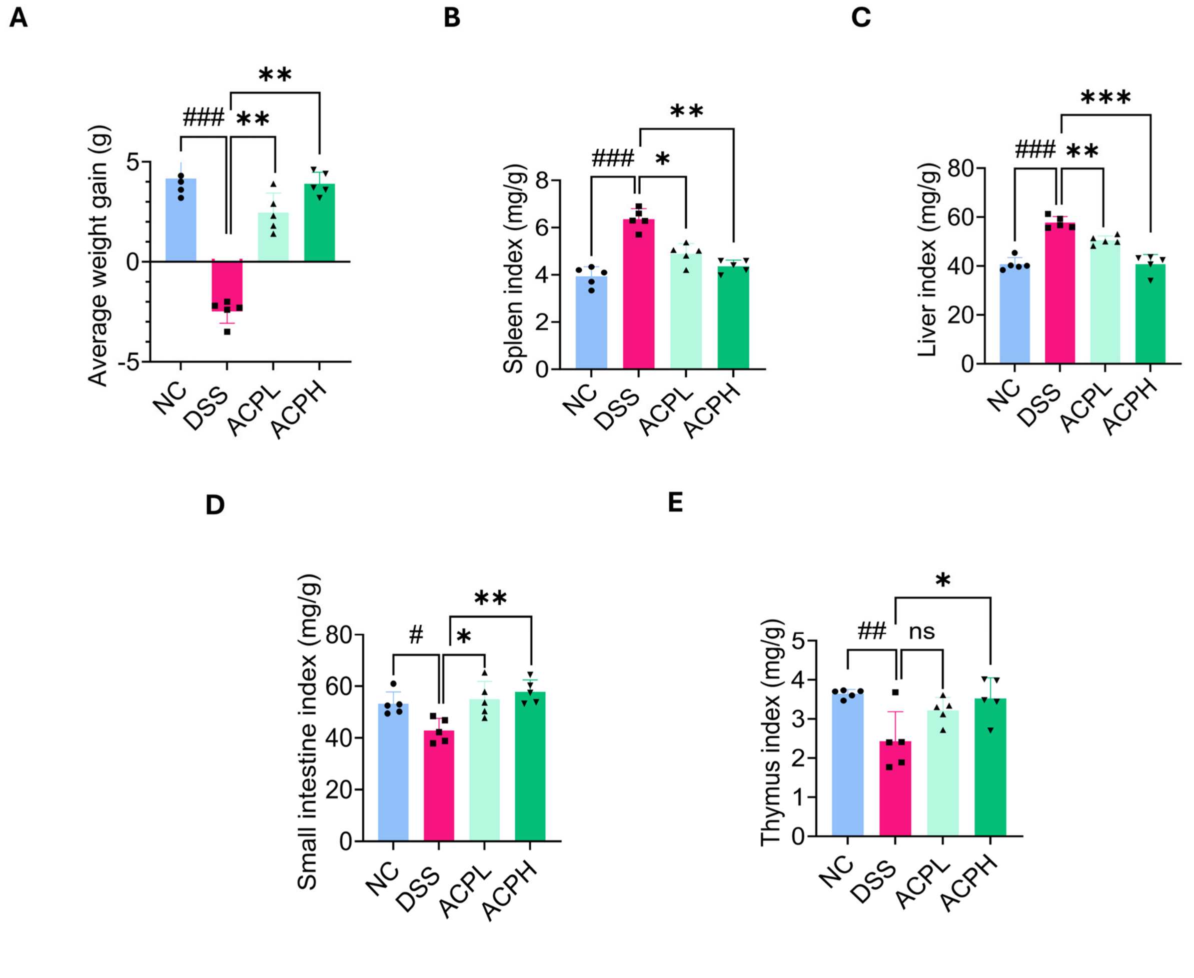

Figure S1. ACP alleviates colitis symptoms**:** (**A**) Average weight gain; (**B**) Spleen index; (**C**) Liver index; (**D**) Small intestine index; (**E**) Thymus index; Results reflected as mean ± SD. # $p < 0.05$, ## $p < 0.01$, ### $p < 0.001$, compared to NC, ns (not significant), * $p < 0.05$, ** $p < 0.01$, and *** $p < 0.001$, compared to the DSS group.

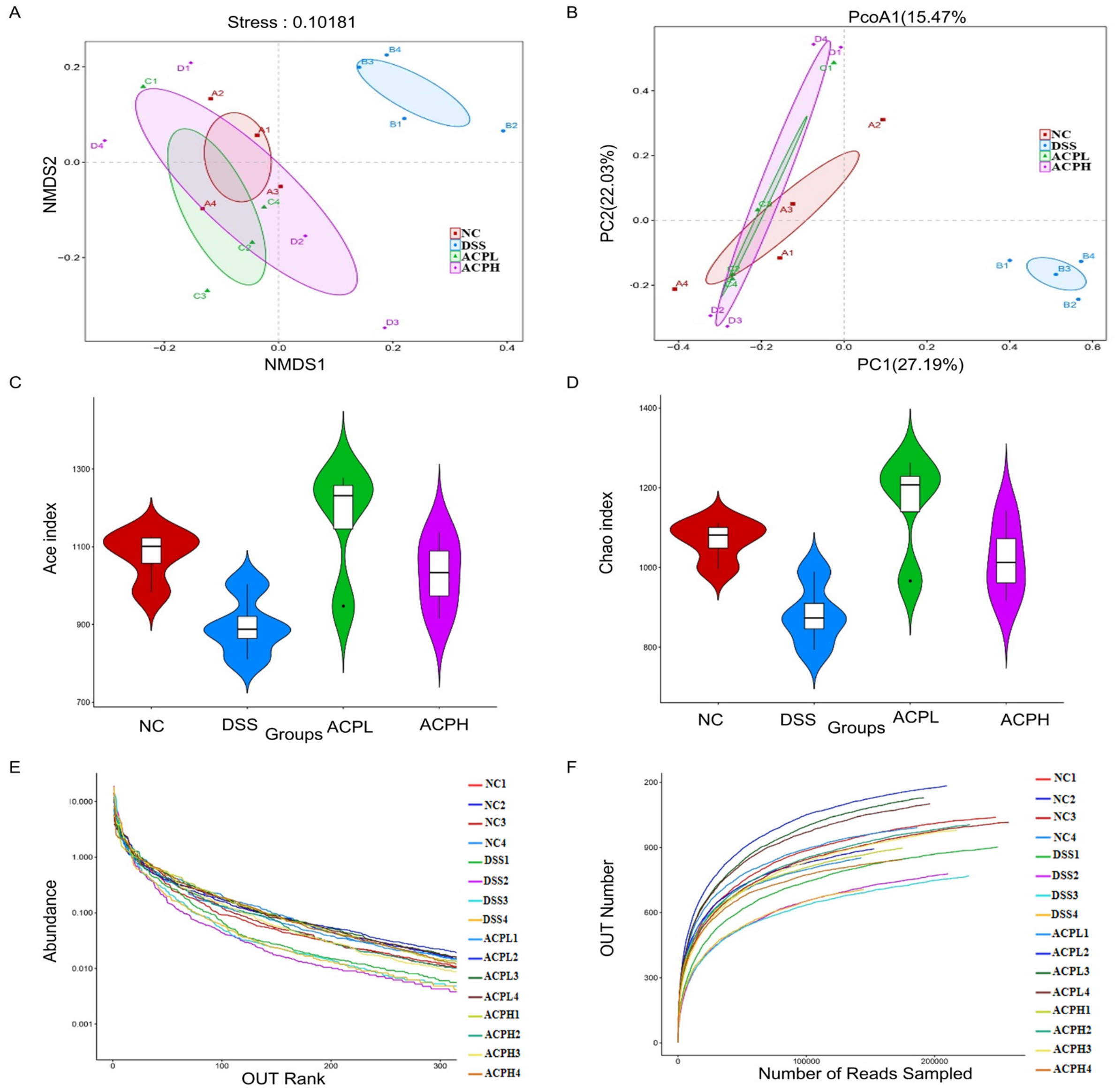

Figure S2. ACP mitigates DSS-induced gut dysbiosis: Beta diversity analysis, including (**A**) Non-metric Multidimensional Scaling (NMDS) and (**B**) Principal Component Analysis (PCA), evaluates microbial community structure. Alpha diversity indices (**C**) ACE and (**D**) Chao-1 assess richness, abundance, and diversity. (**E**) Rank-abundance curves illustrate the relative abundance of microbial taxa across experimental groups. (**F**) Rarefaction curves provide insight into the microbial diversity of the samples.

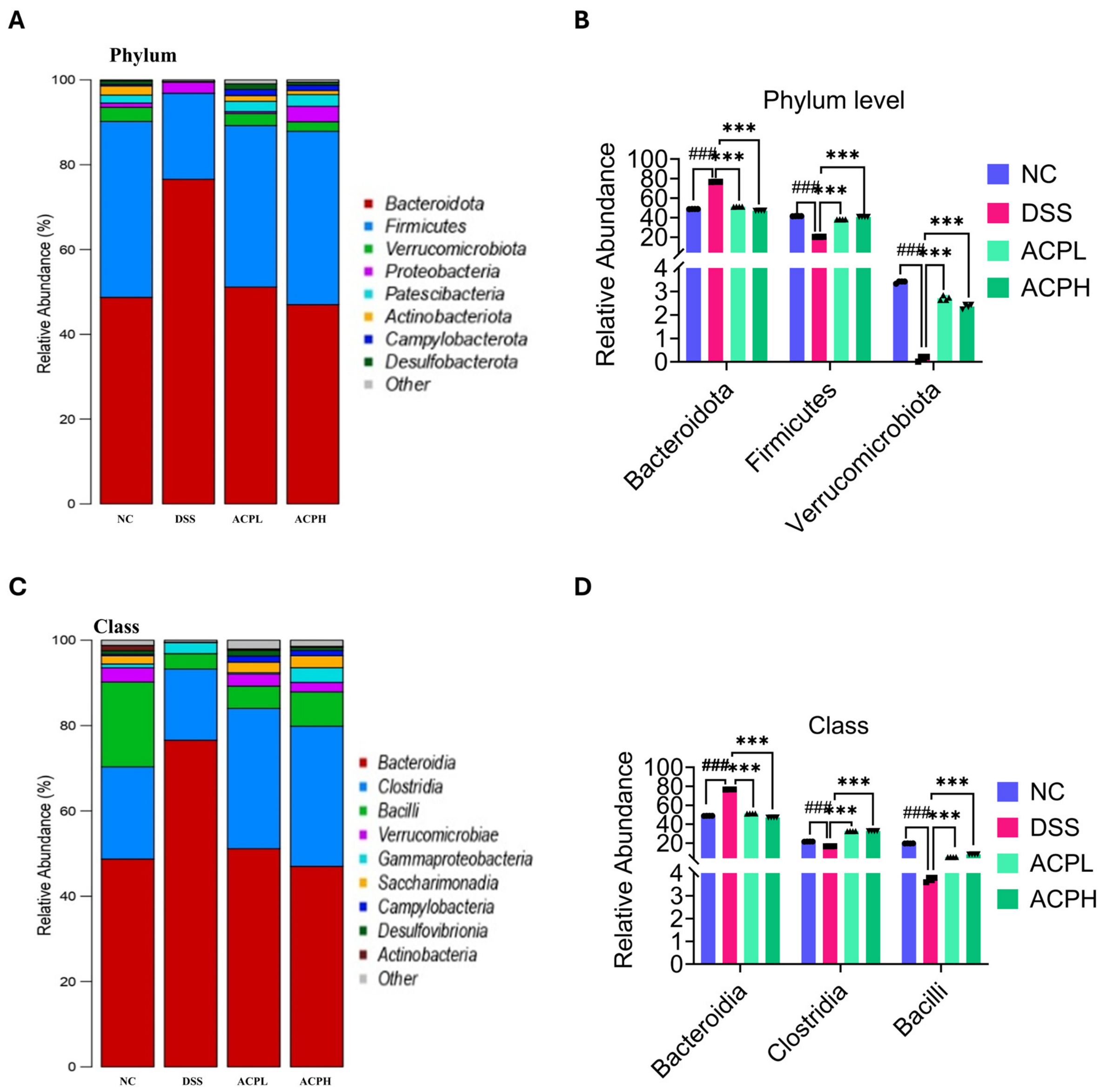

Figure S3. Modulation of gut microbiota ecology by ACP: Microbial diversity at different taxonomic levels: (**A**) Phylum, (**B**) Class. ACP treatment restores microbial balance, enhancing beneficial taxa and mitigating DSS-induced dysbiosis. Results reflected as mean ± SD.### $p < 0.001$, compared to NC, *** $p < 0.001$, compared to the DSS group.

Table S1: Percentage Composition of Bacterial Phylum Across the Different Experimental Groups.

Phylum	NC%	DSS%	ACPL%	ACPH%
Bacteroidota	48.7259041	76.5765267	51.1477631	47.0041302
Firmicutes	41.4657515	20.2724644	38.0911058	40.8737964
Verrucomicrobiota	3.32981241	0.01202721	2.84143743	2.23561489
Proteobacteria	1.00482244	2.63359825	0.44536637	3.65211639
Patescibacteria	1.89313515	0.01659755	2.44303697	2.78357698
Actinobacteriota	2.13301507	0.06181986	1.31868933	0.95116761
Campylobacterota	0.37921845	0.05340081	1.45513339	1.2533831
Desulfobacterota	0.84135397	0.02754231	1.32908121	0.73975511
Other	0.22698696	0.34602284	0.92838644	0.50645934

Table S2: Relative Abundance of Bacterial Class Across the Different Treatment Groups.

Class	NC%	DSS%	ACPL%	ACPH%
Bacteroide	48.7259041	76.5765267	51.1477631	47.0041302
Clostridia	21.6049244	16.6751258	32.8499534	32.8389894
Bacilli	19.8604722	3.59709807	5.24115239	8.03480697
Verrucomicrobiae	3.3269736	0.01202721	2.84062767	2.23096156
Gammaproteobacteria	0.9098404	2.55975118	0.31944915	3.43932046
Saccharimonadia	1.89313515	0.01659755	2.44303697	2.78307392
Campylobacteria	0.37921845	0.05340081	1.45513339	1.2533831
Desulfovibrionia	0.84135397	0.02754231	1.32908121	0.73975511
Actinobacteria	1.24553034	0.02898558	0.31796459	0.24209939
Other	1.2126474	0.45294474	2.05583815	1.43347989

Table S3: Percentage Composition of Bacterial Genus Across the Different Experimental Groups.

Genus	NC%	DSS%	ACPL%	ACPH%
Lachnospiraceae_NK4A136_group	8.378527672	10.3882624	16.32173266	15.4809037
Bacteroides	3.650598576	29.53461912	4.948965063	2.712141944
Lactobacillus	11.91959528	1.765714753	2.394451545	2.223541367
Parabacteroides	1.271671104	9.534571013	1.663510864	1.824989184
Alistipes	3.406815287	1.582059251	3.831635318	3.108933404
Alloprevotella	3.735171624	3.104463538	1.65352386	1.37198036
Akkermansia	3.326973598	0.01202721	2.84062767	2.230961556
Prevotellaceae_UCG-001	3.453182608	0.163329517	2.603368859	1.913528388
Candidatus_Saccharimonas	1.893135152	0.01659755	2.442902007	2.782445091
Ligilactobacillus	2.625549577	0.077936323	0.734719547	2.834889477

Table S4. List of primers used to check mRNA expression level.

Gene	Forward Primer 5' to 3'	Reverse Primer 5' to 3'
TNF-α	GGTGCCTATGTCTCAGCCTCTT	GCCATAGAACTGATGAGAGGGAG
IL-17	GACTCTCCACCGCAATGAAGAC	CTCTTCAGGACCAGGATCTCTTG
IL-18	TCCAACTTCCAGCAGCCATACATC	GGTAGCACTGATCCTTAGCACTGAC
IL- 23	GAGCAACTTCACACCTCCCT	TAGAACTCAGGCTGGGCATC
IL-4	ACCAGGAGCCATATCCACGGATG	GGTGTTCTTCGTTGCTGTGAGGAC
IL-6	TACCACTTCACAAGTCGGAGGC	CTGCAAGTGCATCATCGTTGTTC
Zo-1	GAGCCTAATCTGACCTATGAACC	TGAGGACTCGTATCTGTATGTGG
Occludin	TGAAAGTCCACCTCCTTACAGA	CCGGATAAAAAGAGTACGCTGG
Claudin1	CCAGGTACGAATTTGGTCAGG	TGGTGTTGGGTAAGAGGTTGT
CRP	GATTCCTGAGGCTCCAACACAC	GATTCCTGAGGCTCCAACACAC
TGFB	TGATACGCCTGAGTGGCTGTCT	CACAAGAGCAGTGAGCGCTGAA
FOXP3	CCTGGTTGTGAGAAGGTCTTCG	TGCTCCAGAGACTGCACCACTT
GATA3	CCTCTGGAGGAGGAACGCTAAT	GTTTCGGGTCTGGATGCCTTCT
TLR4	AGCTCCTGACCTTGGTCTTG	CGCAGGGGAACTCAATGAGG
MyD88	ATCGCTGTTCTTGAACCCTCG	CTCACGGTCTAACAAGGCCAG
NF-κB	GAGCTGTGCGGTCTGTTAAAG	GGTAGTTCGGTTCCACACCA
β-actin	GGTCATCACTATTGGCAACG	ACGGATGTCAACGTCACACT
GAPDH	TGTAGACCATGTAGTTGAGGTCA	AGGTCGGTGTGAACGGATTTG
